# Effects of Helicobacter Pylori Infection on Serology and Intestinal Mucosal Changes in Pediatric Patients With Celiac Disease: A Retrospective Cohort Study

**DOI:** 10.7759/cureus.11134

**Published:** 2020-10-24

**Authors:** Sukru Gungor, Ahmet Alpay Köylü

**Affiliations:** 1 Pediatric Gastroenterology Department, Kahramanmaras Sutcu Imam University Medical Faculty, Kahramanmaraş, TUR; 2 Biochemistry Department, Necip Fazıl City Hospital Child Health and Diseases Unit, Kahramanmaraş, TUR

**Keywords:** gluten sensitive enteropathy, gastric intestinal metaplasia, helicobacter pylori

## Abstract

Introduction

Helicobacter pylori (HP) and celiac disease (CD) can cause similar mucosal damage to the duodenal mucosa. For this reason, the relationship between these two diseases has been the subject of research recently. Our study aims to investigate the effects of HP infection on serology and pathology in pediatric patients with CD or potential celiac disease (PCD).

Methods

It is a retrospective cohort study conducted in the third-level education and research hospital between July 2017 and May 2019. The serological and pathological data of patients diagnosed with CD or PCD were compared statistically according to the presence of HP.

Results

An analysis of the histopathological data of the endoscopic biopsy samples showed Helicobacter pylori in eight (50%) of PCD patients and 37 (41.6%) of CD patients. No significant difference was found between the two groups (P=0.531). We found that dokutransglutaminas antibody level (DTG) and endomysium antibody level (EMA) serology decreased significantly after HP eradication therapy in HP (+) PCD (P=0.002, P<0.001). Intestinal metaplasia was not present in PCH. However, intestinal metaplasia was present in five patients (13.5%) with HP (+) CD and two patients (3.8%) with HP (-) CD. However, that difference was not statistically significant between the two groups (P=0.095).

Conclusion

Our study demonstrated that HP may augment CD’s serology and serological improvement is possible after HP treatment particularly in HP (+) PCD. Therefore, we recommend re-perform diagnostic studies after HP treatment before commencing a gluten-free diet in HP (+) suspected CD cases.

## Introduction

Helicobacter pylori (HP) is one of the rare infectious agents associated with the development of various autoimmune disorders as well as causes some gastroduodenal disorders such as chronic gastritis, duodenal ulcers, and adenocarcinoma [1]. Celiac disease (CD) is a common autoimmune disorder of the gut triggered by gluten. Although the pathogenesis of CD is well-defined, its increasing prevalence has led to an investigation of a number of environmental risk factors that can trigger autoimmunity against gluten in the small intestine. Findings about the timing of gluten intake in infant feeding [2], rotavirus infections [3], and cesarean delivery have led to the emergence of opinions that exposure to different microbiota during the perinatal period affects the risk of CD development. [4]. Helicobacter pylori settles in the antrum mucosa of the stomach and reproduces. It may cause pathological mucosal changes (such as ulcer, intraepithelial lymphocytosis, and villous atrophy) in the gastric and duodenum by increasing gastric acid secretion, activating both the Th1/Th17 and T-reg pathways [5-6]. We anticipate that this intestinal mucosal damage (such as intraepithelial lymphocytosis and villous atrophy) caused by Helicobacter pylori may confuse the diagnosis of celiac disease. Therefore, we aimed to retrospectively investigate the effects of HP on CD serology, duodenal mucosal changes, and Marsh classification.

## Materials and methods

This study is a retrospective cohort study examining the clinical, biochemical, and histological data of 105 pediatric patients who were followed up between July 2017 and May 2019 in a tertiary research hospital with a diagnosis of CD or potential CD.

This study was performed in compliance with the Helsinki Declaration. The third level was approved by the education research hospital and the medical school ethics committee.

Serologic evaluation

Antiendomysium (EMA) values were determined with the indirect fluorescence technique using distal monkey esophageal parts mounted on glass slides (EUROIMMUN, Luebeck, Germany). Serum tissue transglutaminase immunoglobulin A (IgA) (dokutransglutaminas antibody level; DTG) levels were quantified by using the ELiA Celikey IgA kit (Phadia AB, Uppsala, Sweden). As recommended by the manufacturer, serum samples containing an antibody titer greater than 20U/mL were considered positive. Total IgA was measured in all patients, and a serum IgA concentration of below 0.07 g/L was considered an IgA deficiency [7].

HLA DQ Type

Deoxyribonucleic acid (DNA) was isolated from a 2 ml blood sample with an automatic DNA isolation device. HLA-DQA1 and HLA-DQB1 alleles and subtypes were typed from this DNA. The sequence-specific primers-polymerase chain reaction (SSP-PCR) method was used for this. Transactions were made according to the protocol specified by the manufacturer.

Endoscopic evaluation

Duodenal biopsy samples were obtained by upper gastrointestinal endoscopy. Three biopsy samples per patient were taken from the distal duodenum. Duodenal ampulla biopsies were routinely obtained since 2010 because the latest studies have suggested that that region may be the only region affected by CD [8].

Patients’ endoscopic mucosal appearances (nodularity, cracked mud appearance) were reviewed from their medical records. The inter-group differences were statistically analyzed.

Histological evaluation

The histopathological data of the patients were reviewed from their medical records. In patients with histopathologically detectable HP, spiral-shaped helicobacteria adhered to the surface epithelium in Giemsa and hematoxylin and eosin (H&E) stained sections were investigated [9]. The signs of intraepithelial lymphocytosis, hyperplastic crypt, villous atrophy, and intestinal metaplasia (in the gastric mucosa) were sought. The inter-group differences were statistically analyzed.

Intraepithelial Lymphocytosis

CD3 positive ≥30 IEL/100 epithelial cells [10].

Intestinal Metaplasia

It is the displacement of the epithelium and gastric mucosa, which histologically resembles the intestinal mucosa and is often associated with chronic atrophic gastritis. Intestinal metaplasia cannot always be classified objectively by histological methods because it is sometimes difficult to recognize the brush border of absorbent cells using conventional H&E staining. In these cases, alcian blue (pH 2.5)/periodic acid-Schiff (AB PAS) and high iron diamine/alcian blue (HID-AB) techniques can be used to identify neutral mucins, sialomucins, and sulfomucins. When these conditions are in question, AB PAS staining was used in our study [11].

The mucosal injury was graded by the standard Marsh-Oberhuber classification [12], where Marsh 0: Normal mucosa; Marsh I: Increased intraepithelial lymphocytosis (> 25/100 enterocytes); Marsh II; Hyperplastic cryptic structures; Marsh III: (a) Partial villous atrophy, (b) Subtotal villous atrophy, (c) Total villous atrophy.

The diagnostic evaluation in celiac disease [13] is as follows:

• Patients with or without symptoms who had a >10-fold increase in DTG and/or EMA and who had a histological mucosal injury grade of Marsh type 3a, 3b, or 3c were diagnosed with CD.

• Patients with normal IgA levels who had symptoms, a >10-fold increase in DTG and/or EMA, HLA DQ2, DQ8 positivity, and a histological grade of Marsh type 2a or greater were diagnosed with CD.

• Patients with an IgA deficiency and clinical symptoms, who had a >10-fold increase in anti-transglutaminase IgG level and/or anti-endomysium IgG level, HLA DQ2/DQ8 positivity, and a histological grade of Marsh type 2a or greater were diagnosed as the CD.

Potential celiac patients

Serological, histological, and biochemical data were analyzed. Patients with genetic (HLA-DQ2/HLA-DQ8), serological (DTG and EMA) positivity for celiac disease but whose histological evaluation was < Marsh type 2 were considered potential celiac disease (PCD) [14].

HP eradication therapy was given to three patients with HP infection whose celiac genetics and antibodies were positive and histologically had March type 2 pattern. These patients were included in the group of potential celiac patients because a significant decrease in EMA and DTG antibodies was observed with HP eradication therapy alone.

Evaluation of nutritional status

The height of participating children younger than two years was measured with the help of an infantometer, with the children in the supine position on a flat surface and their head and knees fixed by a second person. The height of children older than two years was measured using a perpendicular portable stadiometer calibrated to the nearest millimeter, with socks and shoes removed. Their weight was measured with a digital electronic weighing scale to the nearest decimal fraction of one kilogram, with the children wearing light clothing.

Weight Z score, height Z score, height-weight Z score, and body mass index (BMI) Z score were calculated by age and sex using the World Health Organization (WHO) data. Patients with a Z score lower than -2 in any of the body weight, height, and BMI parameters were considered malnourished.

Statistical analyses

Statistical analyses were carried out using the Statistical Package for the Social Sciences for Windows 22 (IBM Corp., Armonk, NY) software package. Study variables were presented as mean ± standard deviation, number (n), and percentage (%). The normality of the distribution of numerical variables was tested using the Kolmogorov-Smirnov test. Normally distributed parameters were analyzed with the student’s t-test or one-way analysis of variance (ANOVA) test; non-normally distributed numerical variables were compared using the Mann Whitney-U test or the Kruskal Wallis test. The chi-square test, student’s t-test, or Mann Whitney-U test was used to test statistical significance. The one-way ANOVA test was used to test the significance of the difference of the arithmetic means of a dependent variable between more than two independent groups. Logistic regression analysis was used to test the relationship between a dependent variable and one or more independent variables. A p-value that was smaller than 0.05 was considered statistically significant.

Inclusion criteria

Laboratory, histological, and genetic data of all patients between the ages of six months and 18 years who underwent endoscopy with suspicion of celiac disease were examined. Patients who met the criteria for celiac disease or potential celiac disease and who were histologically evaluated for HP were included in the study.

## Results

Among 16 patients in the PCD group, 11 (68.8%) were male and five (31.2%) were female. Of 89 patients diagnosed with CD, 62 (68.7%) were female and 27 (30.3%) were male. There was a significant difference between the two groups with respect to sex. There was a male predominance in the PCD group and a female predominance in the CD group (P=0,003). The mean age of the patients was 9.43±4.57 (0.9-18 age). The youngest patient was 0.9 years old. No significant difference existed between the PCD and CD groups with respect to weight, height, BMI Z score, and mean age (P=0,614, P=0.943, P=0.403, P=0.101, respectively). Among the patients with PCD, weight and height Z scores were significantly lower in the HP (-) ones than the HP (+) ones (P=0.037, P=0.044, respectively) (Table 1).

**Table 1:** Evaluation of the clinical findings of the patients * Independent student t-test ** Crosstabs: chi-square test CD: celiac disease. HP: Helicobacter pylori. PCD: potential celiac disease. BMI: body mass index

	PCD (16) Mean± Standard deviation				CD (89) Mean± Standard deviation			
	HP (-) (8)	HP (+) (8)	Toplam	P*	HP (-) (52)	HP (+) (37)	Toplam	P*
Age	8±4.53	7.48±3.40	7.74±3.88	0.802	9.82±4.79	9.62±4.48	9.73±4.64	0.846
Weight Z score	-1.59±0.90	-0.07±1.72	-0.83±1.54	0.044	-1.04±1.63	-1.05±1.49	-1.05±1.56	0.989
Height Z scores	-1.68±1.12	-0.11±1.56	-0.91±1.56	0.037	-0.92±1.48	-0.98±1.41	-0.94±1.43	0.864
BMI Z scores	-0.87±0.98	-0.35±1.55	-0.61±1.28	0.432	-0.81±1.26	-0.82±1.20	-0.81±1.23	0.962
	PCD (16) N-%				CD (89) N-%			
	HP (-) (8)	HP (+) (8)	Toplam	P**	HP (-) (52)	HP (+) (37)	Toplam	P**
Gender Female	5-62.5	0-0	5-31.2	0.007	29-55.8	33-89.2	62-69.7	0.001
Male	3-37.5	8-100	11-68.8		23-44.2	4-10.8	27-30.3	
İnability to gain weight	4-50	1-12.5	5-31.3	106	22-42.3	12-32.4	33.4-38.2	0.345
Short stature	3-37.5	1-12.5	4-25	0.248	21-40.6	11-29.7	32-36	0.302
Diarrhea	1-12.5	1-12.5	2-12.5	1.000	5-9.6	1-2.7	6-6.7	0.200
Abdominal pain	1-12.5	6-75	7-43.8	0.012	25-48.1	19-51.4	44-49.4	0.761
Constipation	1-12.5	2-25	3-18.8	0.522	12-23.1	10-27	22-24.7	0.670

An analysis by admission complaint revealed that abdominal pain, inability to gain weight, and short stature were the most common symptoms, and there was no significant difference between the rates of those symptoms in both groups (P=0.675, P=0.596, P=0.395, respectively). The rate of abdominal pain was greater in HP (+) patients than the HP (-) ones in the potential CD group (P=0.012) (Table 1).

The cracked mud appearance in the endoscopic view of the duodenum and/or bulbus was found in five (31.3%) of PCD patients and 71 (79.8%) of CD patients. This appearance was significantly more prevalent in CD patients than PCD patients (P<0.001).

An analysis of the histopathological data of the endoscopic biopsy samples showed HP in eight (50%) of PCD patients and 37 (41.6%) of CD patients. No significant difference was found between the two groups (P=0.531).

An analysis of the serological and histopathological data of the HP(+) PCD group with regard to Helicobacter presence revealed a DTG level of 78.25±24.52 U/mL prior to HP eradication therapy and 36.12±20.21 U/mL after HP eradication therapy, thus indicating a significant drop in the DTG level in the post-treatment period compared to the pre-treatment period (P=0.002). The HP (-) PCD group had a DTG level of 66.53±20.69 U/mL. The DTG level was not significantly different between the two groups with respect to the presence of HP (P=0.306) (Table 2).

**Table 2:** Evaluation of serological and histopathological data according to the presence of Helicobacter pylori in potential celiac patients * Independent student t-test ** Crosstabs: chi-square test CD: celiac disease. HP: Helicobacter pylori. BHPT: before HP eradication therapy. AHPT: after HP eradication therapy. DTG: dokutransglutaminas antibody level. EMA: endomysium antibody level

	HP (+) (8)			HP (-) (8)		P*
DTG U/mL	78.25±24.52			66.53±20.69		0.306
EMA U/mL	80.09±29.57			63.62±20.83		0.219
	HP(+) N-%			HP (-) N-%		P**
DTG >100 U/mL	4-50			1-12.5		0.141
EMA >100 U/mL	3-37.5			1-12.5		0.248
	BHPT	AHPT	P	First value	Control	P*
DTG U/mL	78.25±24.52	36.12±20.21	0.002	63.62±20.83	51.43±22.74	0.282
EMA U/mL	80.09±29.57	25.25±17.64	<0.001	66.53±20.69	47.37±22.93	0.101
	Histopathological evaluation N-%					P**
Marsh Tip 0	0-0			6-75		0.001
Marsh Tip 1	5-62.5			2-25		
Marsh Tip 2	3-37.5			0-0		
Marsh Tip 3	0-0			0-0		
Intestinal Metaplasia	0-0			0-0		

EMA was 80.09±29.57 U/mL before HP eradication therapy and 25.25±17.64 U/mL after the HP eradication therapy in the HP (+) PCD. The EMA level was significantly lowered after the therapy (P<0.001). In the HP (-) PCD group, on the other hand, the EMA level was 63.62±20.83 U/mL. The EMA level was not significantly different between the two groups based on HP positivity in the PCD patients (P=0.219) (Table 2).

In the HP (-) PCD group, the DTG level at the time of diagnosis was 63.62±20.83 U/mL while the control DTG level without diet modification after three months was 51.43±22.74 U/mL. There was no significant difference between the diet-free control level and the initial DTG level (P=0.282). The EMA level was 66.53±20.69 U/mL at the time of diagnosis and 47.37±22.93 U/mL three months later. No significant difference was detected between the control and initial EMA levels (P=0.101) (Table 2).

The histopathological evaluation of HP (+) PCD revealed that five patients (62.5%) had Marsh Type 1 injury and three (37.5%) had Marsh Type 2 injury. In Helicobacter pylori (-) PCD, six (75%) patients had Marsh Type 0 injury while two (25%) had Marsh Type 1 injury. A significant difference was found between HP (+) and (-) PCD patients P=0.001).

A comparison of DTG and EMA levels by the presence of HP showed that DTG levels were significantly higher in HP (+) CD patients as compared to HP(-) ones (P=0.027).

Although EMA was higher in HP (+) CD patients as compared to HP (-) ones, the difference did not reach statistical significance (P=0.081) (Table 3).

**Table 3:** Evaluation of serological and histopathological data according to the presence of Helicobacter pylori in celiac patients * Independent student's t-test ** Crosstabs: chi-square test CD: celiac disease. HP: Helicobacter pylori. DTG: dokutransglutaminas antibody level. EMA: endomysium antibody level

	HP (+) (37)				HP (-) (52)		P*
DTG	268.85±64.95				236.34±68.62		0.027
EMA	267.22±65.20				241.37±69.56		0.081
	HP (+) (37) N-%				HP (-) (52) N-%		P**
DTG>100	36-97.3				49-94.2		0.491
EMA>100	37-100				49-96.1		0.223
	HP (+) (37)				HP (-) (52)		
	Before gluten-free diet CD	After gluten-free diet CD	P		Pre diet CD	Post diet CD	P*
DTG	268.85±64.95	67.73±49.02	<0.001		236.34±68.62	51.46±43.42	<0.001
EMA	267.21±65.20	49.09±38.97	<0.001		241.37±69.56	44.35±32.10	<0.001
	Histopathological evaluation N-%						P**
	HP (+) (37) N-%			HP (-) (52) N-%			
Marsh Tip 0	0-0			0-0			0.018
Marsh Tip 1	0-0			0-0			
Marsh Tip 2	1-2.7			9-17.3			
Marsh Tip 3	36-97.3			43-82.7			
Intestinal Metaplasia	5-13.5			2-3.8			0.095

In Helicobacter pylori (+) CD, the before gluten-free diet DTG and EMA levels were significantly higher than the after gluten-free diet levels (P<0.001, P<0.001, respectively) (Table 3).

In Helicobacter pylori (-) CD, the before gluten-free diet DTG and EMA levels were significantly higher than the after gluten-free diet (P<0.001, P<0.001, respectively) (Table 3).

The histopathological examination of HP (+) CD showed that 36 patients had (97.3%) Marsh type 3 injury while one patient (2.5%) had Marsh type 2 injury. In HP (-) CD, 43 patients (82.7%) had Marsh type 3 and nine patients had Marsh type 2 (18.4%) injuries. There was a significant difference between HP (+) and (-) CD in terms of histopathological evaluation (P=0.018) (Table 3).

An evaluation on the basis of histopathological intestinal metaplasia development demonstrated that there was no intestinal metaplasia in PCD while it was present in five (13.5%) patients with HP (+) CD and two (3.8%) patients with HP (-) CD. When intestinal metaplasia was evaluated between HP (+) CD and HP (-) CD, it was shown that it was more common in HP (+) patients. However, that difference was not statistically significant between the two groups (P=0.095) (Table 3).

## Discussion

Most studies in the literature that assessed the relationship between celiac disease and HP have been conducted between the CD and non-CD control groups. Our study, on the other hand, is a retrospective work that was distinctly performed between PCD and CD and assessed the effects of HP on celiac serology and histopathology.

The combined prevalence of HP infection among children, adults, and all populations is estimated to be 42% (41-44%), 62% (61-64%), and 54% (53-55%), respectively [15]. Consistent with the literature, HP was detected in 45 (42.8%) of the children included in our study.

Histological data gained from some HP prevalence studies in celiac patients suggest that there may be a link between HP infection and CD [16]. In a study by Konturek et al., the HP prevalence was higher in CD patients as compared with the control group [17]. Jozefczuk et al., on the other hand, could not demonstrate any higher HP prevalence in CD than in the general population [18]. There are also some studies that suggest that HP infection prevents CD development [19-20]. We did not come across any study that compared PCD and CD by the presence of HP. However, in line with previous reports, we failed to demonstrate any difference between PCD and CD in terms of HP prevalence (P:0.531). In agreement with our study, literature studies have not shown any difference in symptomatology between HP (+) and HP (-) CD [1,19,21]. However, we detected a significantly higher rate of abdominal pain in HP (+) PCD than HP (-) PCD (P=0.0012). This may be attributed to a lower number of PCD patients in addition to the abdominal pain intensifier effect of HP.

Narang et al. showed a higher number of patients with a DTG level >100 among HP (+) CD patients than the HP (-) ones (P=0.003) [19]. Unlike our results, previous studies have shown trivial non-significant differences between the DTG serum levels of HP (+) and HP (-) patients. Nevertheless, they reported mildly elevated antigliadin levels in HP (+) patients [1,21]. We likewise found significantly higher DTG levels in HP (+) CD than HP(-) CD (P=0.027). We also showed that DTG and EMA levels were significantly lowered after HP eradication treatment in the HP (+) PCD group that was not administered any diet for celiac disease (P:0.002, P<0.001, respectively). This suggests that HP may be a relevant factor in increasing DTG and EMA levels.

European Society of Pediatric Gastroenterology Hepatology and Nutrition (ESPGHAN) guidelines for the diagnosis of celiac disease in children were revised in 2012 [22]. Accordingly, it was stated that when symptomatic children with DTG and EMA titers more than >10 times greater than the normal limit have an HLA-DQ2/DQ8 phenotype, CD can be diagnosed with serology alone, without making a pathology evaluation. A subsequent study by ESPGHAN has recommended a biopsy in order to reduce the risk of false positives among patients who are DTG positive but have DTG titers lower than 10 times the normal limit [22-23]. It was also stressed that a biopsy is not an essential criterion for a serology-based diagnosis when an HLA test is available and a patient is symptomatic. However, none of the above-mentioned studies have mentioned any relationship of HP infection with PCD or CD. In our study, in agreement with the literature, DTG and EMA titers were >10 times greater than normal in patients diagnosed with CD who were started on a diet (mean 249.85±68.67 and 252.24±68.59, respectively). We performed a histological evaluation for all patients. According to the Marsh classification, 10 (20%) patients were Marsh type 2 and 79 patients were Marsh type 3. We performed a genetic evaluation for all potential celiac patients and all patients in the Marsh type 2 classification. We did not perform it in the other CD, as it was an expensive test. Potential celiac patients genetically had one of the HLA-DQ2/DQ8 haplotypes. DTG and EMA titers were lower at the time of the first biopsy in a majority of patients than in celiac patients with atrophy, as reported in other studies [24]. Tosco et al. reported that the DTG and EMA titers of some patients were negative or showed fluctuations during the follow-up of PCD. They stressed that this condition was more common among patients with lower DTG or EMA titers at baseline [25]. However, there is no information as to the presence of HP in PCD. Our study also demonstrated that EMA and DTG levels were lower in PCD than CD with atrophy. We showed a significant improvement in DTG and EMA titers after HP treatment as compared to pre-treatment titers (P=0.002, P<0.001, respectively). Although we could not fully explain its reason, this condition indicates that HP influences CD serology.

There may occur diagnostic difficulties with the histological evaluation of celiac type caused by gastrointestinal pathogens [26-28]. Histological features involve both inflammatory (increased intraepithelial lymphocytes) and intestinal epithelial architectural alterations (crypt hyperplasia and villous atrophy). Previous data have indicated that duodenal intraepithelial lymphocytosis with villous structures (Marsh I lesion) is a relatively common finding in duodenal biopsies [29], and it has been reported that, in addition to lymphocytic duodenitis, duodenal morphological injury caused by HP is also involved in the potential etiology. It has been reported that Helicobacter pylori is associated with a number of ulcers and causes architectural alterations in the duodenal mucosa such as non-specific duodenal ulcers, villous atrophy, and crypt hyperplasia [30]. However, information about distal duodenal alterations among HP-infected patients is scarce. Jinga et al. reported two cases with inflammation and crypt hyperplasia in the distal duodenum mimicking CD in HP-infected adults [30].

When we evaluated HP’s effect on histopathology in PCD and CD, we detected a more severe duodenal injury (Marsh types 1, 2) in HP (+) PCD than HP (-) PCD (P=0.001).

Similarly, there was a more severe mucosal injury (Marsh types 2, 3) in HP (+) CD than HP (-) CD (P=0.018). Also, there was no intestinal metaplasia in potential CD while we detected a greater severity of intestinal metaplasia in HP (+) CD than HP (-) CD, albeit this difference was not statistically significant (P=0.095). We do not exactly know if this resulted from CD and/or HP. However, we are of the opinion that it may be linked to genetic factors, HP’s virulence, and immune pathology brought about by CD and/or HP. There is a need for more comprehensive and larger studies to test these possibilities. The limitations of our study are a low number of subjects and a lack of demonstration of pathological improvement among patients with serological improvement after HP treatment.

Our study supports the serology-based instructions for diagnosing CD, which was issued by ESPGHAN. In suspected cases, there are gray zones of treatment and follow-up with respect to HP (+) and HP (-) status of symptomatic cases with DTG and EMA titers that are <10 times lower. Our study sheds light on these aspects of the disease.

## Conclusions

In conclusion, our study demonstrated that HP may augment CD’s serology and serological improvement is possible after HP treatment, particularly in HP (+) PCD. Therefore, we recommend re-performing diagnostic studies after HP treatment before commencing a gluten-free diet in HP (+) suspected CD cases. Furthermore, the prevalence of intestinal metaplasia in HP (+) CD seems worthy of study.
